# Short-Term Drop in Antibody Titer after the Third Dose of SARS-CoV-2 BNT162b2 Vaccine in Adults

**DOI:** 10.3390/vaccines10050805

**Published:** 2022-05-20

**Authors:** Jonas Herzberg, Bastian Fischer, Heiko Becher, Ann-Kristin Becker, Human Honarpisheh, Salman Yousuf Guraya, Tim Strate, Cornelius Knabbe

**Affiliations:** 1 Department of Surgery, Krankenhaus Reinbek St. Adolf-Stift, 21465 Reinbek, Germany; human.honarpisheh@krankenhaus-reinbek.de (H.H.); tim.strate@krankenhaus-reinbek.de (T.S.); 2 Institut für Laboratoriums-und Transfusionsmedizin, Herz-und Diabeteszentrum Nordrhein-Westfalen, Universitätsklinik der Ruhr-Universität Bochum, 32545 Bad Oeynhausen, Germany; bfischer@hdz-nrw.de (B.F.); cknabbe@hdz-nrw.de (C.K.); 3 Institute of Medical Biometry and Epidemiology, University Medical Center Hamburg-Eppendorf, 20246 Hamburg, Germany; h.becher@uke.de; 4 Abteilung für Psychiatrie und Psychotherapie, Asklepios Klinik Harburg, 21075 Hamburg, Germany; annk.becker@asklepios.com; 5 Clinical Sciences Department, College of Medicine, University of Sharjah, Sharjah P.O. Box 27272, United Arab Emirates; salmanguraya@gmail.com

**Keywords:** SARS-CoV-2, humoral immunity, antibody longevity, vaccination, BNT162b2

## Abstract

Little is known about the longevity of antibodies after a third dose of the mRNA-based SARS-CoV-2 vaccine BNT162b2 (BioNTech/Pfizer, Mainz, Germany). Therefore, serum antibody levels were evaluated after a third dose of BNT162b2 in healthy adult healthcare workers in Germany. These antibody levels dropped significantly within a short period of 11 weeks from 4155.59 ± 2373.65 BAU/mL to 2389.10 ± 1433.90 BAU/mL, *p*-value < 0.001 but remained higher than after the second dose (611.92 ± 450.31 BAU/mL). To evaluate the quality of the humoral immune response, we additionally measured neutralizing antibodies, which also showed a small but significant decrease within this short period. These data underline the positive effect of a third dose of BNT162b2 concerning antibody re-induction but also shows a drop of Anti-SARS-CoV-2-IgG within a short span of time.

## 1. Introduction

Vaccination is intended to be a core element in the global fight against the COVID-19 pandemic. Therefore, a variety of vaccines were approved rapidly. The protective role of mRNA-based SARS-CoV-2 BNT162b2 vaccine, as introduced by BioNTech/Pfizer, has been endorsed by several studies [1,2]. The rapidly increasing number of COVID-19 cases among the previously vaccinated individuals has led to the addition of a third dose of vaccines as a booster [3,4].The immediate immune response after the third dose has been evaluated by a wide range of clinical trials [5,6,7,8]. Until now, there has been a lack of best clinical evidence about the durability and long-term impact of the immune response that is invariably induced by the third dose.

Foregoing in view, we assessed the anti-spike-IgG-antibody-titers and neutralizing antibodies, 4 and 11 weeks after the third dose of BNT162b2 as well as 11 weeks after the administration of the second dose of BNT162b2 within a previously elaborated study cohort.

## 2. Materials and Methods

### 2.1. Study Population

We included employees of the Hospital Reinbek St. Adolf-Stift, a German secondary care hospital to the longitudinal ProCoV-study, established in April 2020 [9]. In the follow-up analysis, all participants with a homogeneous prime-boost-protocol, who had received three doses of BNT162b2, were invited to participate in this study. Participants with a previous self-reported SARS-CoV-2-infection within the last six months before the last blood drawn were excluded.

All participants provided a blood sample 11 weeks after the second dose of vaccinate and then 4 weeks after the third dose between November and December 2021. Subsequently, in January 2022, a follow-up blood sample was drawn 11 weeks after the third dose.

The study was approved in April 2020 by the ethics committee of the medical association Schleswig-Holstein, Germany and all participants provided written and informed consent prior to inclusion. This trial was prospectively registered in the German Clinical Trial Register (DRKS00021270).

### 2.2. Anti-SARS-CoV-2-IgG Antibodies

Anti-SARS-CoV-2-IgG antibodies were measured using the anti-SARS-CoV-2- assay (IgG) from Abbott (Chicago, IL, USA) and the values were quantitatively expressed in binding-antibody-units per mL (BAU/mL). Values below 7.1 BAU/mL were determined seronegative whereas values above 7.1 BAU/mL were graded positive, as mentioned by the manufacturer.

### 2.3. Surrogate Virus Neutralization Test

To detect neutralizing antibodies, a novel enzyme-linked immunosorbent assay (ELISA)-based surrogate virus neutralization test was used instead of a cell-culture based neutralization test, as this showed a good correlation and can easily be performed in routine laboratories [10]. With this, neutralizing anti-SARS-CoV-2 antibodies were detected according to the instructions of the manufacturer (NeutraLISA™ SARS-CoV-2 Neutralization Antibody Detection KIT (Euroimmun, Lübeck, Germany)). In brief, controls and plasma samples were diluted 1:10 and subsequently mixed with HRP-conjugated RBD. After incubation (37 °C, 30 min) samples were transferred to a 96-well ELISA-plate precoated with recombinant ACE2 antigens. After incubating the plate (15 min, 37 °C) and removing the supernatant, a substrate solution was added to each well. The reaction was stopped by adding a stop-solution to each well. Finally, adsorption was read at 450 nm. Results are presented in percentages of binding inhibition using the following formula: Binding inhibition [%] = (1−(OD450 (sample)/OD450 (blank)) × 100.

Values above 35% were considered positive, between 20% and 35% equivocal and those below 20% were graded negative in accordance to the manufacturer’s instructions.

### 2.4. Statistical Analysis

The statistical analysis was done using the IBM SPSS Statistics Version 25 (IBM Co., Armonk, NY, USA) and GraphPad Prism 9 (GraphPad Software, CA, USA).

All variables were presented as means with standard deviation and medians with interquartile range. Categorical variables were shown as numbers with percentages. The differences between groups were analyzed using the Wilcoxon test. A *p*-value < 0.05 was considered statistically significant.

## 3. Results

Overall, 153 participants fulfilled the inclusion criteria, of which 103 provided a blood sample 11 weeks after the third dose. Six participants were excluded from this cohort due to a reported SARS-CoV-2-infection within the last six months. The characteristics of the recruited 97 participants are shown in Table 1.

At 11 weeks after the second dose, 93 participants (95.9%) showed a positive antibody response with a mean titer of 611.92 ± 450.31 BAU/mL. At 4 weeks after the third dose, all participants showed a seropositive result and remained seropositive 11 weeks after the third dose.

A comparison of the antibody titer 11 weeks after second dose with those 11 weeks after the third dose showed a significant rise in antibody titer that was induced by the third dose (611.92 ± 450.31 vs. 2389.10 ± 1433.90, *p*-value < 0.001). A corresponding rise in the neutralizing antibodies (83.26 ± 27.79 vs. 99.51 ± 0.64, *p*-value < 0.001) was also reported.

**Figure 1:**
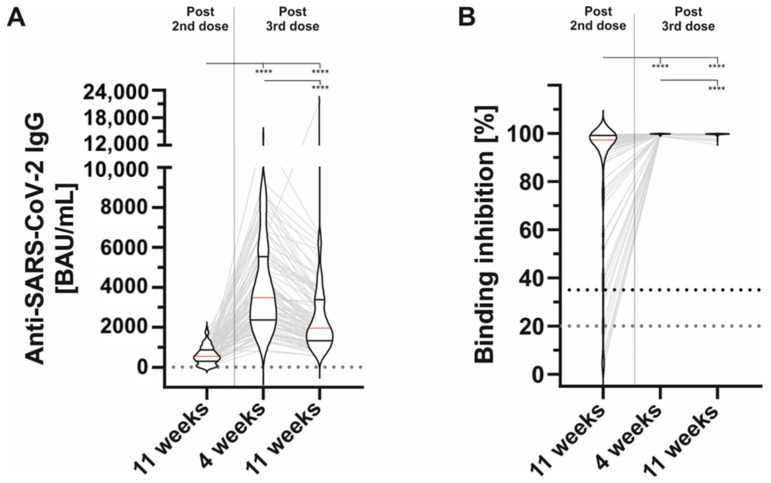
The vaccine induced serological immune response at different timepoints post second and third doses of BNT162b2. (**A**): Anti-SARS-CoV-2-IgG at different timepoints (left: 11 weeks post second vaccine dose, right: 4 and 11 weeks after third vaccine dose, respectively). (**B**): Binding inhibition at different timepoints (left: 11 weeks post second vaccine dose, right: 4 and 11 weeks after third vaccine dose, respectively). Horizontal lines within plotted data show the median (red line) and interquartile ranges. **** *p* < 0.0001 (Mann–Whitney U-test).

Interestingly, the mean IgG-titer four weeks after the third dose was found to be 4155.59 ± 2373.65 BAU/mL which dropped significantly to 2389.10 ± 1433.90 BAU/mL 11 weeks after the third dose (*p*-value < 0.001) (Table 2, Figure 1A). We also noted a slight but significant drop concerning the inhibition capability of neutralizing antibodies. However, neutralizing antibodies remained at a high level 11 weeks after the third dose (99.73% ± 0.18% vs. 99.51, *p*-value < 0.001) (Figure 1B).

## 4. Discussion

Our study demonstrates a significant rise in the binding-antibody titer and the neutralizing antibodies following the third dose as compared to the serological response to the second dose of BNT162b2.

On the other hand, our data has reported a rapid and significant drop in the binding-antibody titers and neutralizing antibody capabilities in a short term between the fourth and elevens week after third vaccination, though the titers remain high. The detected minimal drop in neutralizing antibodies after the third vaccination are in line with previous data for the second vaccination dose. It has been shown that neutralizing antibodies are much more stable over time after the second BNT162b2 vaccination than anti-SARS-CoV-2 IgG binding antibodies [11].

Until now, little has been known about the sustained and long-term impact of the vaccination-induced immune response. Data after two doses of BNT162b2 showed a significant decrease within six to nine months [12,13]. Recently published data about the short-term immunological effect of the third dose of BNT162b2 showed a significant increase of antibodies within the first weeks [14,15,16].

Despite the small cohort size of our study, several other studies have shown positive inductive immunological effect of a third dose of BNT162b [5,6,8]. Our study provides an original clinical data about the antibody persistence of the second and third dose during a short-term follow-up. While persistence of the humoral immunity is much stronger after the administration of the third dose of BNT162b2, the dropping antibody titers following the third dose signals the potential necessity of a fourth dose of vaccine. This possibility has been recently suggested by some reports [17,18].

Until now, little is known about the correlation of humoral immunity and the severity of COVID-19 disease. A study by Feng et al. suggests that a vaccine-induced SARS-CoV-2 antibody concentration of 264 (95% CI: 108, 806) BAU/mL is suitable to provide 80% protection against symptomatic infection [19]. However, the data refer to the viral alpha variant (B.1.1.7) and further studies are needed.

### Limitations

This study is limited by its small sample size and single-center design. This study was based on a longitudinal evaluation of hospital employees which led to a lack of diversity. Unfortunately, no data about the cellular immunity are available within this follow-up. As often seen in studies including hospital employees, women are relatively overrepresented [20]. In addition, there is a lack for participants under higher risk such as elderly people. The use of an ELISA-based surrogate assay instead of cell-culture based assay might limit the comparability, even though some studies have proven a good correlation [10]. Unfortunately, no data about the cellular immunity are available within this follow-up. Further studies are needed to evaluate the infection rate depending on several antibody titers which can detect potential measurable thresholds.

## 5. Conclusions

This cohort study provides the first human-based clinical data about the longevity of the humoral immune response induced by a third dose of BNT162b2 against COVID-19. This data may potentially facilitate determination of the necessity of an additional fourth dose of anti-COVID-19-vaccine.

## Figures and Tables

**Table 1:** Baseline characteristics of the study cohort (*n* = 97).

Characteristics	Mean ± SD	Median	IQR
Age (M ± SD; years)	48.03 ± 9.45	48.00	15
BMI (M ± SD; kg/m^2^)	25.61 ± 4.71	24.69	6.16
Sex	*n* (%)		
Women (%)	73 (75.3)		
Men (%)	24 (24.7)		
Comorbidities	*n* (%)		
Cardiac (%)	20 (20.62)		
Pulmonary (%)	9 (9.28)		
Metabolic (%)	18 (18.56)		
Immunologic (%)	1 (1.03)		
Other (%)	18 (18.56)		
Smoking (%)	25 (25.77)		

M: mean. IQR: Interquartile range. SD: standard deviation. BMI: body mass index.

**Table 2:** Antibody titers and binding inhibition capabilities of neutralizing antibodies at different timepoints within the study cohort (*n* = 97).

	Mean ± SD	Median	IQR
Immunoglobulin G			
A1:11 weeks after second dose (BAU/mL)	611.92 ± 450.31	543.60	599.90
B1: 4 weeks after third dose (BAU/mL)	4155.59 ± 2373.65	3482.70	3200.80
C1: 11 weeks after third dose (BAU/mL)	2389.10 ± 1433.90	1949.90	1921.40
Difference C1-A1	1777.18 ± 1470.51	1466.80	2075.00
Difference C1-B1	−1766.49 ± 1329.81	−1574.60	1440.05
Binding inhibition			
A2: 11 weeks after second dose (%)	83.26 ± 27.79	97.23	14.80
B2: 4 weeks after third dose (%)	99.73 ± 0.18	99.76	0.10
C2: 11 weeks after third dose (%)	99.51 ± 0.64	99.69	0.23
Difference C2-A2	16.25 ± 27.61	2.29	14.95
Difference C2-B2	−0.22 ± 0.51	−0.10	0.23

M: mean. SD: standard deviation. IQR: Interquartile range. BMI: body mass index. BAU: binding antibody units. A1: IgG 11 weeks after second dose. B1: IgG 4 weeks after third dose. C1: IgG 11 weeks after third dose. A2: binding inhibition 11 weeks after second dose. B2: binding inhibition 4 weeks after third dose. C2: binding inhibition 11 weeks after third dose.

## Data Availability

The raw data supporting the conclusions of this article will be made available by the authors on reasonable request, without undue reservation.
